# The Effect of Diclofenac Sodium-Loaded Poly(Lactide-co-Glycolide) Rods on Bone Formation and Inflammation: A Histological and Histomorphometric Study in the Femora of Rats

**DOI:** 10.3390/mi11121098

**Published:** 2020-12-12

**Authors:** Karoline M. Reich, Petrus Viitanen, Ehsanul Hoque Apu, Stefan Tangl, Nureddin Ashammakhi

**Affiliations:** 1 Karl Donath Laboratory for Hard Tissue and Biomaterial Research, Division of Oral Surgery, University Clinic of Dentistry, Medical University of Vienna, 1090 Vienna, Austria; karoline.reich@meduniwien.ac.at; 2 Austrian Cluster for Tissue Regeneration, 1200 Vienna, Austria; 3 Institute of Biomaterials, Tampere University of Technology, 33101 Tampere, Finland; petrus.viitanen@bioretec.com; 4 Laboratory of Developmental Biology, Faculty of Biochemistry and Molecular Medicine, University of Oulu, 90220 Oulu, Finland; hoqueapu@msu.edu; 5 Institute for Quantitative Health Science and Engineering, Department of Biomedical Engineering, Michigan State University, East Lansing, MI 48824, USA; 6 Division of Plastic Surgery, Department of Surgery, Oulu University Hospital, 90220 Oulu, Finland; 7 Center for Minimally Invasive Therapeutics (C-MIT), University of California, Los Angeles, CA 90095, USA; 8 Department of Bioengineering, University of California, Los Angeles, CA 90095, USA; 9 Department of Biomedical Engineering, Michigan State University, East Lansing, MI 48824, USA

**Keywords:** biodegradable, inflammation, foreign body reaction, multi-nucleated giant cells, non-steroidal anti-inflammatory drug

## Abstract

Implants made of poly(lactide-co-glycolide) (PLGA) are biodegradable and frequently provoke foreign body reactions (FBR) in the host tissue. In order to modulate the inflammatory response of the host tissue, PLGA implants can be loaded with anti-inflammatory drugs. The aim of this study was to analyze the impact of PLGA 80/20 rods loaded with the diclofenac sodium (DS) on local tissue reactions in the femur of rats. Special emphasis was put on bone regeneration and the presence of multinucleated giant cells (MGCs) associated with FBR. PLGA 80/20 alone and PLGA 80/20 combined with DS was extruded into rods. PLGA rods loaded with DS (PLGA+DS) were implanted into the femora of 18 rats. Eighteen control rats received unloaded PLGA rods. The follow-up period was of 3, 6 and 12 weeks. Each group comprised of six rats. Peri-implant tissue reactions were histologically and histomorphometrically evaluated. The implantation of PLGA and PLGA+DS8 rods induced the formation of a layer of newly formed bone islands parallel to the contour of the implants. PLGA+DS rods tended to reduce the presence of multi-nucleated giant cells (MGCs) at the implant surface. Although it is known that the systemic administration of DS is associated with compromised bone healing, the local release of DS via PLGA rods did not have negative effects on bone regeneration in the femora of rats throughout 12 weeks.

## 1. Introduction

Biocompatible, biostable materials such as metals have been used in traumatology and orthopedic surgery for a long time [1]. However, in certain cases, especially in pediatric surgery, the removal of such implants is required [2,3,4,5], which necessitates a second operation [6]. Therefore, biodegradable implants with good mechanical properties and biocompatibility have been developed in the last few decades [7,8,9]. The copolymers of polylactide (PLA) and polyglycolide (PGA) such as poly(lactide-co-glycolide) (PLGA), have been among the most favorable biodegradable implants because of their predictable biodegradation rate, as well as their modifiable mechanical and physicochemical properties such as stiffness, elastic modulus and viscoelastic properties [10,11,12,13]. PLGA has been utilized thus far to produce pins, rods, screws, tacks, wire, scaffolds and membranes [11,12,14,15].

In the body, PLGA is degraded via a hydrolytic scission of its ester bonds into the monomers, lactic acid (LA) and glycolic acid (GA), which are subsequently metabolized via the tricarboxylic acid (TCA) cycle to generate CO_2_ and H_2_O, and some of GA may be excreted in the urine [11,13,16,17]. However, during their degradation process, resulting particles and products lead to the stimulation of the immune system and lead to the development of local chronic inflammation [18,19,20,21].

Although acute inflammation is a natural response of the host to trauma [22] and as such an important phase in the wound and fracture healing, its extension beyond one week [21], to persist as chronic inflammation [21] is not ideal and can lead to problems such as osteolysis [20,23], fluid accumulation swelling, burst or sinus formation [18,24]. It is characterized by the infiltration of the area by inflammatory cells, namely macrophages and multinucleated giant cells, the formation of granulation tissue and later of a fibrous capsule that shuts the foreign material off from the body [18,19,20,21]. In extreme cases, surgical invention is required, adding to the risks and costs of the treatment. In general, the prevalence of foreign body reactions (FBR) to biodegradable implants ranges from 0.7% up to 7.9%, depending on the type of the polymer and its degradation rate, implantation site, fixation mode as well as patient-associated factors such as age, immune situation and chronic disease [6,19,25,26,27,28].

On this basis, approaches to immunomodulate tissue reactions to biodegradable implants are undertaken. For this purpose, multifunctional implants that locally release anti-inflammatory agents can be utilized [29]. Glucocorticoids such as dexamethasone, nonsteroidal anti-inflammatory drugs (NSAIDS), antibiotics, bone morphogenetic proteins etc. have already been used to locally control tissue reactions in the surroundings of implanted PLGA device [30,31,32,33,34,35]. Not all anti-inflammatory agents can withstand harsh conditions of implant processing methods such as high temperature, which is needed to melt the polymer. In the case of PLGA, melting temperature is 160.7 °C [36]. In addition, the selected anti-inflammatory agent should have well documented clinical safety and efficacy profile.

Although there are many such agents, it was found that among the non-cyclo-oxygenase (COX) specific inhibitor anti-inflammatory agents, diclofenac sodium (DS) is the most specific. Moreover, DS is one of the best-studied and most effective non-steroidal anti-inflammatory drugs (NSAIDs) for the clinical treatment of inflammation of various musculoskeletal conditions and injuries worldwide. DS is well-tolerated in the body. It acts by inhibiting the synthesis of prostaglandins, which are important mediators of inflammation. Since DS is characterized by a melting temperature of 296 °C [37], well above the melting temperature of PLGA, it was selected as the agent of choice to achieve immunomodulation in this study. DS loaded osteosynthesis implants have been recently developed by our team and their drug release properties have been optimized [37,38].

The aim of this study was to evaluate the potential of diclofenac sodium containing poly(lactide-co-glycolide) implants for providing an immunomodulatory response without having a negative impact on bone formation. Because the anti-inflammatory effect of DS is mediated through the inhibition of prostaglandins, which are known to play a crucial role in bone metabolism, the investigation of these implants in the bone is of significant importance.

It was thus necessary to answer two central questions that comprised the basis of this study. (1) Does the local release of DS have an effect on inflammation and FBR? (2) Since inflammation is a key prerequisite for bone healing, does the local release of DS compromise bone healing? To answer these questions, semi-automatic histomorphometry and histological analysis were used to study the effect of local DS release from PLGA rods that were implanted in the femora of rats for 3, 6 and 12 weeks.

## 2. Materials and Methods

### 2.1. Implant Material

Rod-shaped implants were prepared from a commercially available copolymer of lactide and glycolide (PLGA) 80/20 (Purasorb^®^, Purac Biochem B.V., Gorinchem, The Netherlands). Diclofenac sodium (DS, Sigma-Aldrich, Espoo, Finland) was compounded with the matrix polymer, and a small-scale laboratory twin-screw extruder was used to produce self-reinforced (SR) rods having a diameter of 2.0 mm and they were cut into 15-mm long implants. The fabrication method is described in greater detail elsewhere [37]. The final concentration of DS in the implants was defined to be 8% of the dry weight (8 wt.%) in the DS containing rods (SR-PLGA+DS8). DS was chosen because of its good clinical record and high melting temperature (Tm) of 296 °C [37] which allows melt processing at the required temperatures of below 200 °C. The concentration of 8 wt.% DS was chosen based on our previous studies which measured the release pattern of the PLGA (80/20) implants and where this concentration showed the best release properties [37,39].

Self-reinforced PLGA (SR-PLGA) rods having no DS were also used to serve as a control. The rods were sterilized by gamma irradiation at a minimum dose of 2.5 mrad. Packed sterile implant packages were opened in the operation room. The SR-PLGA and SR-PLGA+DS8 implants were manufactured in the Institute of Biomaterials, Tampere University of Technology, Tampere, Finland.

### 2.2. Surgery

**Figure 1:**
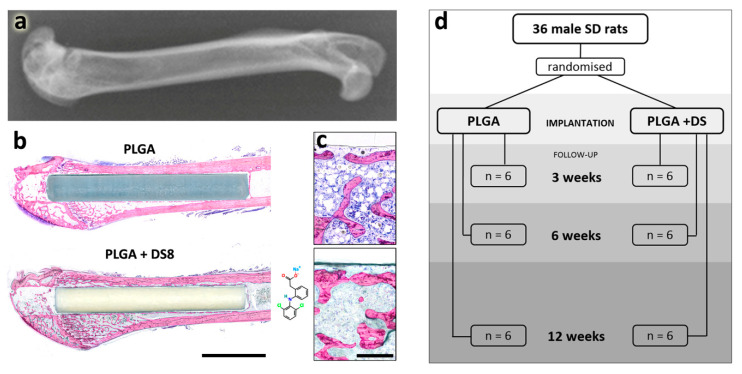
Experimental setup. (**a**) Experiments were conducted in the femur of rats: X-ray illustrating the anatomical region. (**b**) Poly(lactide-co-glycolide) (PLGA) and PLGA+DS8 rods were implanted in the femoral shaft via a drill hole created in the intercondylar space of the femur. Scale bar, 5 mm. (**c**) Magnified images of new bone formation in (**b**), scale bar, 300 µm. (**d**) illustrates the experimental setup: number of rats assigned to PLGA and PLGA+DS8 groups and respective follow-up periods.

Thirty-six male Sprague–Dawley rats with a mean age of 14.1 weeks (SD 3.2) and a mean weight of 378 g (SD 43) were operated on. Rats were randomly divided into two groups, 18 rats received PLGA+DS8 implants, the other 18 rats DS-free PLGA implants. Study endpoints were 3, 6 and 12 weeks, with six rats in each subgroup (Figure 1). The rats were anaesthetized by isoflurane inhalation (isoflurane 4.5% plus oxygen 1.5 lpm during induction, and isoflurane 2.5% plus oxygen 0.5 lpm for maintenance). All animals received a single dose of cefuroxime 20 mg/kg s.c. 15 min before surgery.

The right knee of the rat was shaved and cleaned with 80% alcohol. A parapatellar incision of 2 cm was made on the medial side of the knee. The patella was dislocated laterally, and the distal head of the femur was exposed. A drill hole, with a diameter of 2 mm, was made through the intercondylar space along the axis of the femur. A PLGA rod (15 mm length and 2 mm diameter) was introduced through the drill hole. The rod was tapped into the level of the articular surface of the intercondylar notch to allow free mobility of the knee joint. The wound was closed in layers with 4-0 PGA sutures (Dexon^®^, Davis and Geck, Gosport, UK). Analgesia was provided postoperatively using s.c. 0.3 mg/kg injections of buprenorphine (Temgesic^®^, Reckitt and Colman Pharmaceuticals, Inc., Richmond, England) that were administered immediately after the operations and at the first postoperative day.

Postoperatively, the animals were returned to their cages to recover from the anesthesia. Rats were given regular pelleted rat food and tap water ad libitum. After the wounds healed, rats were kept in cages in groups of 4–6 rats in an artificially illuminated room, with 12 h light/12 h darkness. The rats were euthanized after 3, 6, and 12 weeks postoperatively using carbon dioxide.

The study was approved by the Animal Research Committee of the University of Oulu, Finland, and by the Provincial Administrative Board, according to Finnish law. The guidelines of the Ethics Committee of the Oulu University Experimental Animal Centre for the care and use of experimental animals were observed.

### 2.3. Evaluation Methods

#### 2.3.1. Histology

After euthanasia, the right femur was disarticulated, dissected and macroscopically checked for the correct location of the implanted rod and for the possible presence of infection.

The distal half of the femurs was cut and fixed in 4% phosphate-buffered formaldehyde. After dehydration in a series of increasing ethanol concentrations, the specimens were embedded in a light-curing resin (Technovit 7200 VCL^®^ + BPO; Kulzer& Co., Wehrheim, Germany). Undecalcified thin ground sections were prepared according to the Cutting and Grinding Technique described by Donath [40]. The section plane was oriented in the anteroposterior direction, parallel to the longitudinal axis of the femoral shaft, thereby cutting the implanted rod lengthwise in half.

The final sections were stained with Levai Laczko dye [41], and digitized with a digital camera (DXM1200; Nikon Cooperation, Tokyo, Japan) mounted on a Microphot FXA microscope (Nikon Cooperation) at a resolution of 452 pixel/mm (1 pixel equals 2.21653 µm). Single images were assembled to create large overview pictures (Lucia G 4.71, Laboratory Imaging Ltd., Praha, Czech Republic).

In five samples the section plane did not run exactly in the mid-sagittal plane through the center of the rod. Therefore, additional thin ground sections from the second half of the embedded specimen blocks were prepared. These five new sections were scanned using an Olympus BX61VS digital virtual microscopy system (DotSlide 2.4; Olympus, Japan, Tokyo) with a 20× objective resulting in a resolution of 3125 pixel/mm (1 pixel equals 0.32 μm). The resulting differences in the scanning resolutions were taken into account in all further calculations.

A qualitative histological evaluation focusing on bone regeneration, foreign body reaction at the bone-implant interface and the degradation of the implanted rods was performed. Due to the distinct affinity of different tissue types to the staining solution, old and new bone, cartilage, soft/fibrous tissue and PLGA can reliably be distinguished.

#### 2.3.2. Histomorphometry

**Figure 2:**
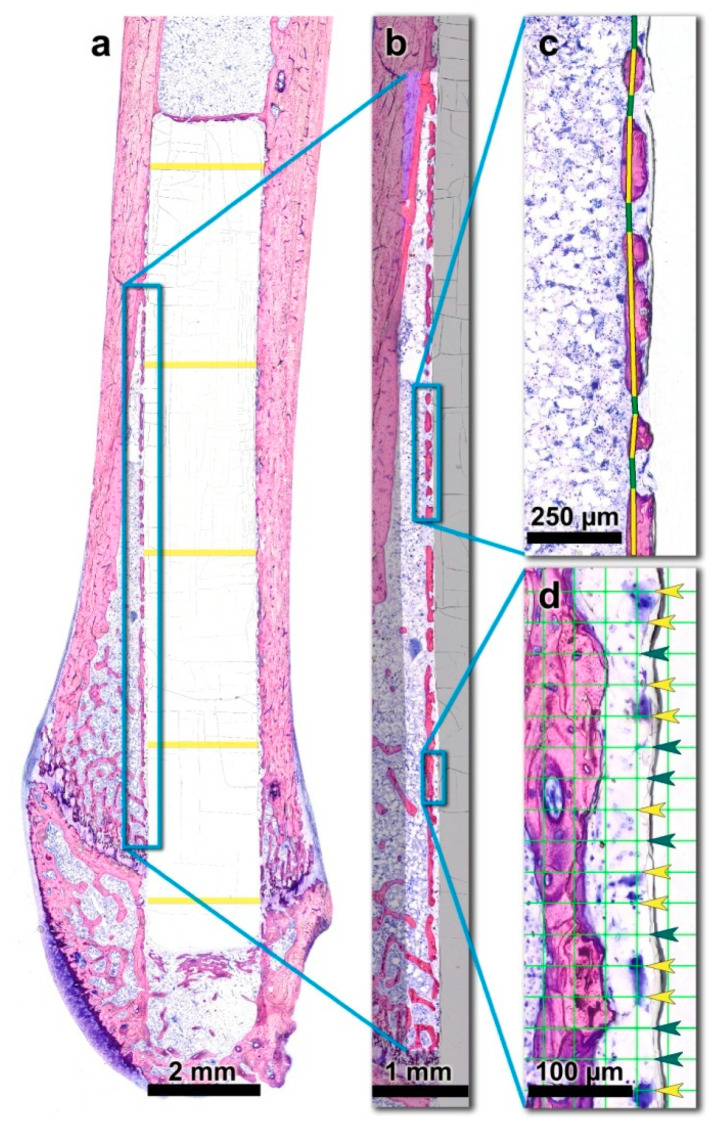
Histomorphometric measurements were based on the histological thin-ground sections of the right femora. (**a**) The region of interest (ROI) is indicated by the blue rectangle. The diameter of the PLGA implant was measured at five predefined, evenly distributed sites over the length of the rods (yellow lines) and averaged per individual. (**b**) New bone volume fraction (nBV/TV) was assessed within the indicated ROI which was segmented into new bone tissue (red), old bone tissue (blue) and soft tissue/medullary space. (**c**) Peri-implant bone capsule (piBC) was defined as the percentage of the rod’s surface that is surrounded by newly formed bone. New bone was marked with a yellow line, interspersing soft tissue with a green line. (**d**) The presence of peri-implant multinucleated giant cells (piMGCs) was assessed with a point counting grid (grid line distance: 30 µm) as the percentage of the rod surface that is lined by MGCs (Yellow arrowheads: grid nodes with an underlying MGC, green arrowheads: intermediate grid nodes).

For histomorphometric analysis, the digitized sections were further processed with Adobe Photoshop^®^ (CS5, Adobe, San Jose, CA, USA). Originally, two regions of interest (ROIs) were set alongside the anterior and posterior bone-implant interface (Figure 2a). Therefore, a rectangular field with the predefined width of 332.5 µm was positioned in the longitudinal direction of the femoral shaft, adjacent to the outer anterior and posterior surface of the PLGA rod, respectively. The length of the rectangles differed depending on the actual position of the PLGA implant within each individual femur. Distally, the ROI was bounded by the cartilaginous epiphysis of the distal femur. Proximally, the endpoint of the ROI was determined as the point where no newly formed peri-implant bone layer was interspersed between the implant and the old compact bone of the femoral shaft. Proximal to this point, the implant is enclosed by the cortex of the diaphysis only. In other words, the transition of the medullary space into the cortex of the diaphysis within the 332.5 µm high rectangle represented the proximal boundary of the ROI (Figure 2a). Based on this definition, the anterior ROI was easily standardizable. However, the posterior region was too irregular and inconsistent to allow for the accurate, commensurable histomorphometric measurement. To avoid bias, only the anterior ROI was measured (and hereinafter referred to as ROI).

The morphometric software Definiens Developer^®^/Definiens Developer XD^®^ (6.0.3/2.7, Definiens AG, Munich, Germany) was used to automatically segment and classify the different tissue types within the ROI. Therefore, an algorithm was developed considering the shape and color of all segments as well as the relationship to other neighboring segments. In this way, old and newly formed bone, cartilage, soft/fibrous tissue, PLGA rod and background could be identified. (Details regarding the segmentation algorithm are provided in the Supplementary material.) Falsely classified areas were manually corrected under microscopic control using Adobe Photoshop software (Adobe, San Jose, CA, USA). The investigator who performed the histomorphometric analysis was blinded to the type of implant animals received.

In order to quantify the possible immunomodulatory effect of DS containing PLGA rods on inflammatory tissue reactions and bone regeneration, the following histomorphometric parameters were calculated using Definiens Developer^®^ (Definiens AG, Munich, Germany) and Adobe Photoshop software (Adobe, San Jose, CA, USA).

*New bone volume per tissue volume (nBV/TV)*

New bone volume fraction (nBV/TV) was defined as the percentage of the newly formed bone volume within the ROI, i.e., within the total tissue volume (TV) comprising new bone, old bone, cartilage and soft/fibrous tissue. nBV/TV was assessed using Definiens Developer 6.0.3^®^/Definiens Developer XD 2.7^®^ (Definiens AG, Munich, Germany) (Figure 2b).

*Peri-implant bone capsule (piBC)*

While nBV/TV is a parameter that quantifies the volume of newly formed bone within a given ROI, it provides no information about the distribution of the new bone within the region. Based on the histological examination, the newly formed bone was found to form a layer of bone islands parallel to the contour of the PLGA rods. This peri-implant bone capsule surrounded the implants like a fenestrated envelope. This observation recalls a well-established standard parameter used in implant histomorphometry, i.e., the bone-to-implant contact (BIC). In contrast to BIC, which assesses the percentage of the implant surface that is in direct contact with newly formed bone, the observed present peri-implant bone capsule mostly runs parallel to the rod surface and is only sporadically in direct contact with it.

In order to quantify this phenomenon, a new parameter termed peri-implant bone capsule (piBC) was defined as the percentage of the rod surface that is surrounded by newly formed bone. This measurement was performed using Adobe Photoshop software (Adobe, San Jose, CA, USA). Within the ROI, a continuous line was drawn to mark the newly formed bone of this “capsule”, and interspersing soft tissue in two different colors. The fraction of the length of the “new bone lines” in relation to the total length of all line segments (“new bone lines” plus “interspersing soft tissue lines”) was calculated (Figure 2c).

*Peri-implant multinucleated giant cells (piMGCs)*

Multinucleated giant cells (MGCs) are a part of the chronic foreign body reaction that frequently occurs after the implantation of biomaterials. They derive from fusing macrophages and are considered as indicators of pathological chronic inflammation [42].

In order to gather quantitative information about the inflammatory processes at the implant-to-tissue interface, the presence of MGC was assessed. Using Adobe Photoshop (Adobe, San Jose, CA, USA), the ROI was overlaid with a point counting grid, a standard instrument of histomorphometry [43,44,45]. Grid nodes had a distance of 30 µm from each other. The size of MGCs was reported to vary greatly between 40 and 200 µm [46].

At each grid node, a dot was drawn either in green, where MGCs were underlying or in yellow where no MGCs were underlying. To determine the percentage of the implant surface that is surrounded by MGCs, the number of MGC-dots was divided by the total number of all dots (“MGCs dots” plus “no-MGCs dots”) and multiplied by 100 (Figure 2d).

*Implant diameter (I.Dm)*

PLGA degrades via a hydrolytic process over time. In addition, MGCs were reported to participate in PLGA engulfment and elimination [21,26]. To assess the dimensions of the PLGA rod in the treatment groups after 3, 6 and 12 weeks, the diameter of the implant was measured manually using the measuring tool in Adobe Photoshop (Adobe, San Jose, CA, USA). PLGA rod diameter was measured at 5 predefined, evenly distributed sites over the length of the rods and averaged per individual. (Figure 2a)

### 2.4. Statistics

Descriptive statistics (Mean, SD, minimum, median, maximum) are performed. Scatterplots are shown for all variables, including mean and a 95% confidence interval for the mean based on the bootstrap. For inference on all variables, we used generalized linear models with gaussian error distributions and a logarithmic link, including Treatment and Week as well as their interaction as independent variables. ANOVA-type tests by means of likelihood ratio tests for the main and the interaction effect were calculated, post-hoc tests were corrected for multiple testing using the single-step method as described by Hothorn et al. [47]. All computations were done using R version 3.6.1 [48].

## 3. Results

### 3.1. Macroscopic Results

Both the PLGA and PLGA+DS8 implant types were similar to handle, and no breaking or bending of any implant occurred. All wounds healed uneventfully, and all rats reached the planned follow-up period. When harvesting the specimens, the anatomical structure of the operated knees seemed normal. There were no microscopic or macroscopic signs of complications such as erythema, swelling, infection etc., neither during the healing period (3/6/12 weeks), nor at the end of the study.

### 3.2. Histological Findings

#### 3.2.1. PLGA and PLGA+DS Implants

All PLGA and PLGA+DS8 implants were located in the correct position in the femoral shaft of all rats as they were placed originally and preserved their structural integrity in all animals. There was no apparent visible reduction in the size or shape of biodegradable implants during the treatment period of 12 weeks. Evidence of degradation was limited to slight surface alterations appearing ridged and in some cases slightly fringed. In some specimens, tiny superficial parts of the PLGA/PLGA+DS8 material appeared to have become detached or scaled off, and cells or bone filled/infiltrated the resulting interspaces in the biomaterial. In general, the degradation of rods was minimal at 3, 6 and 12 weeks postoperatively. Fissures in the rods tended to be slightly more frequently seen at 12 weeks of implantation. The surface condition of the rods seemed not to be related to the presence of DS in the implant.

In several samples, numerous cracks in the embedded implants and occasionally also at the implant-to-tissue intersection were visible. These cracks most probably originate from strain in the PLGA/PLGA+DS8 rods occurring during the specimen preparation process and did not impede the histomorphometric evaluation of the samples.

#### 3.2.2. New Bone Formation

**Figure 3:**
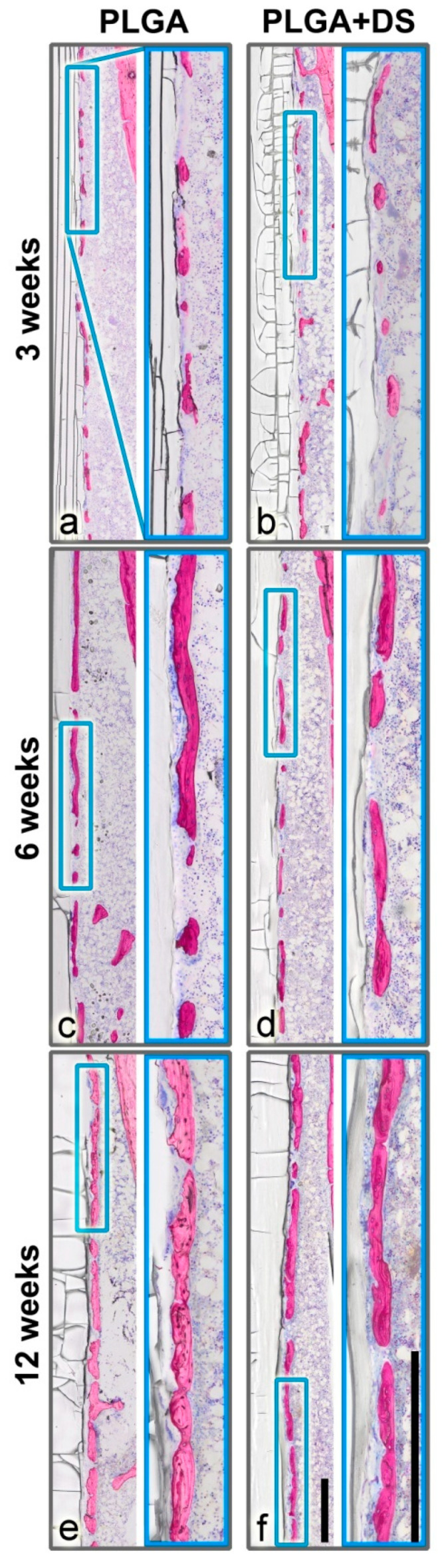
Peri-implant bone capsule. A layer of new bone was formed in close vicinity to the PLGA rods, in all samples. (**a**,**b**) After 3 weeks, only small islands were lining the implant surface. (**c**,**d**) After 6 weeks, the bone islands tended to increase in length and thickness. (**e**,**f**) After 12 weeks, the bone capsule covered the most part of the implant forming a fenestrated capsule in the medullary space. The piBC, i.e., the percentage of the rod surface that is surrounded by newly formed bone, tended to be higher in PLGA groups than in PLGA+DS8 groups. However, new bone volume fraction (nBV/TV) did not differ between the PLGA and PLGA+DS8 groups. Scale bar in overview and magnification images, 500 µm.

In all samples, a layer of new bone was formed in close vicinity to the PLGA implants lining the implant surface like a fenestrated capsule, in the medullary space (Figure 3). The distance between these newly formed “bone islands” and the implant was on average 5–50 µm. Only sporadically, bone islands were in direct contact with the rods. The thickness of these bone trabeculae tended to increase over time from 25 µm at 3 weeks up to 90 µm at 12 weeks postoperatively. Similarly, the length of the largest bone islands increased with time from 50–700 µm in the 3 weeks group up to 2.5 mm in the 12-week groups. However, large and small bone islands co-existed in all groups and the size of the gaps in-between the bone islands varied greatly. This might be indicative of a three-dimensional “net-like envelope”.

Mineralized cartilage embedded into newly formed woven bone was found in the vicinity of the epiphyses. In the 3-week groups, the peri-implant bone capsule was comprised predominantly of woven bone. Particularly in the 6- and 12-week groups, a compaction of woven bone resulting in plexiform bone and remodeling into lamellar bone was observed.

#### 3.2.3. Cells at the Implant Surface

**Figure 4:**
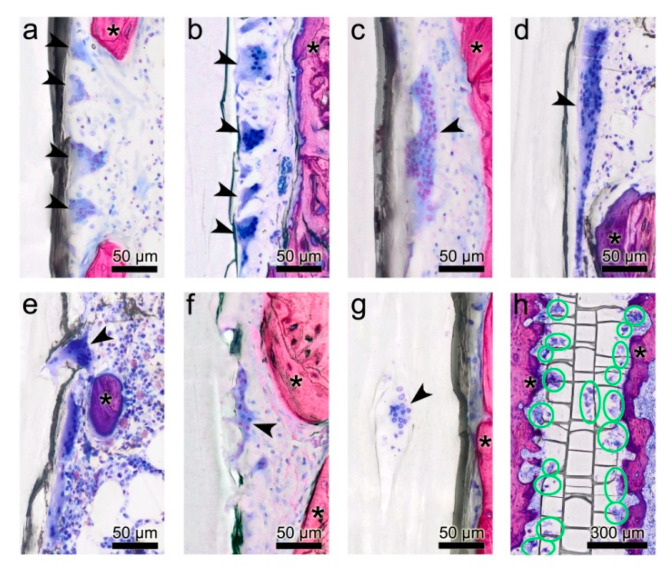
Morphological diversity of multinucleated giant cells (MGCs). (**a**–**d**) MGCs of various shape, size, numbers of nuclei and different staining intensities adhere to the surface of the poly(lactide-co-glycolide) (PLGA) implant in both, the PLGA and PLGA+DS8 groups. (d) Large MGC with a length of 270 µm. (**e**,**f**) MGCs submerge into the PLGA implant by extruding filopodia. (**g**) MGC located in a lacuna within the PLGA implant. (**h**) Overview section parallel to the midsagittal plane of a PLGA implant illustrating the location and activity of MGCs below the surface of a PLGA+DS8 rod 6 weeks after surgery (green circles indicate MGCS). Arrowheads point to multinucleated giant cells; Asterisks indicate bone.

The surface of the PLGA rods was frequently lined by varying numbers of large MGCs. These cells being regarded as an indicator of foreign body reaction, were present in all samples of both types of treatment (PLGA with or without DS) at 3, 6 and 12 weeks. The number of the MGC nuclei countable in the two-dimensional section ranged from at least 6 up to 150 being heterogeneously distributed over the cytoplasm. The total size of MGCs ranged from approximately 30 up to 450 µm. Large MGCs seemed to be slightly more common in rats with longer treatment duration. The shape of the MGCs varied considerably ranging from elongated, flat, to oval-shaped and irregular-shaped with spiky, irregular pseudopodia. Furthermore, the staining intensity of the cytoplasm and of the nuclei was very different among the MGCs. Very light blue MGCs were mixed with dark blue MGCs of various sizes and shapes in individual samples (Figure 4a–h).

MGCs were located in direct contact with the surface of the PLGA rods. Frequently, they were found in pits of the rod surface, resembling resorption lacunae of the bone, or in fissures of the implant most probably resulting from PLGA degradation (Figure 4e–g).

Large MGCs were less commonly seen in PLGA+DS8 groups than in plain PLGA groups, irrespective of the duration of treatment. No significant differences concerning shape or staining behavior could be detected between the PLGA and PLGA+DS8 groups.

#### 3.2.4. Inflammation

Apart from MGCs, there were no other signs of chronic inflammation in terms of a foreign body reaction to the PLGA or PLGA+DS8 implants. No evidence of tissue swelling, sinus formation, or osteolysis was found in any of the samples in either group. Whether the presence of an osseous capsule should be regarded as a type of FBR is controversially discussed [18,19,20,21,49].

### 3.3. Histomorphometry

#### 3.3.1. New Bone Volume per Tissue Volume (nBV/TV)

**Figure 5:**
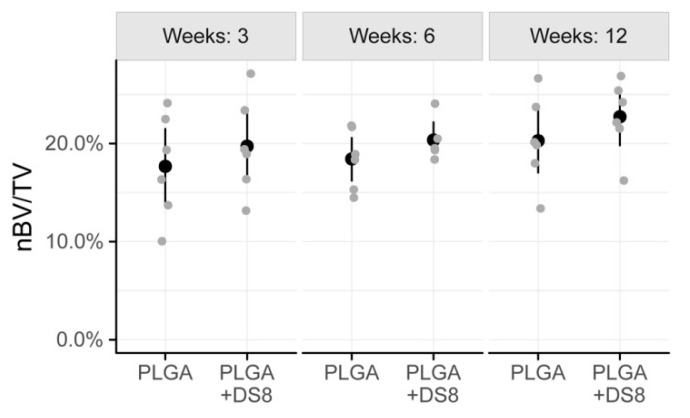
Scatter plot of nBV/TV, i.e., the percentage of newly formed bone volume (nBV) within the tissue volume (TV). (Black dots refer to mean values of the respective groups).

After a healing period of 3 weeks, 17.7% (SD 5.4) of the region of interest was occupied by newly formed bone in the SR-PLGA group and 19.7% (SD 5.0) in the PLGA+DS8 group. After 6 weeks, BV/TV was 18.4% (SD 3.1) and 19.6% (SD 2.6) in the SR-PLGA group and the PLGA+DS8 group, respectively. After 12 weeks, the amount of BV/TV was higher, with 20.3% (SD 4.6) in the PLGA group and 22.7% (SD 3.8) in the PLGA+DS8 group (Figure 5).

There was a slight increase of nBV/TV over time, in both PLGA and the PLGA+DS8 groups. However, the time-dependent changes were not statistically different for the PLGA group and the PLGA+DS8 group (*p* = 0.196). There was no statistically significant difference in nBV/TV between the PLGA and the PLGA+DS8 groups at any time point (*p* = 0.167).

#### 3.3.2. Peri-Implant Bone Capsule (piBC)

While nBV/TV quantifies the total volume of newly formed bone within the ROI, piBC measures the percentage of the rod surface that is surrounded by newly formed bone.

**Figure 6:**
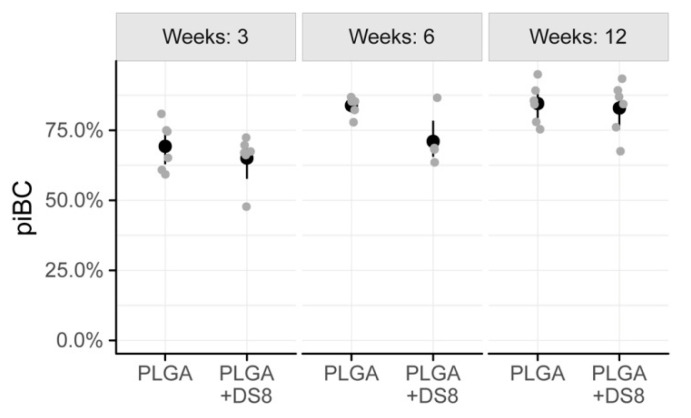
Scatter plot of peri-implant bone capsule (piBC), i.e., the percentage of the rod surface that is surrounded by newly formed bone. (Black dots refer to mean values of the respective groups).

After a healing period of 3 weeks, piBC was 69.3% (SD 8.7) in the PLGA group and 65.1% (SD 8.8) in the PLGA+DS8 group. After 6 weeks, 83.9% (SD 3.3) of the implant surface was lined by newly formed bone in the PLGA group, but only 72.8% (SD 9.1) in the PLGA+DS8 group. After 12 weeks, piBC was 84.6% (SD 7.2) and 82.9% (SD 9.5) in the PLGA and the PLGA+DS8 group, respectively. (Figure 6)

In both the PLGA and the PLGA+DS8 groups, piBC significantly increased over time (*p* < 0.001). A more detailed comparison of the differences between the time points analyzed is listed in Table 1. In addition, there was a significant difference in piBC between the PLGA and the PLGA+DS8 groups (*p* = 0.03); however, post hoc tests revealed that no significant difference exists in concrete comparisons.

#### 3.3.3. Peri-Implant Multinucleated Giant Cells (piMGCs)

In one specimen (of the PLGA+DS8 6 weeks group), the implant-to bone interface was difficult to assess due to massive cracks in the PLGA rod resulting in dark shadows in this region. Therefore, this specimen had to be excluded from the MGCs evaluation.

**Figure 7:**
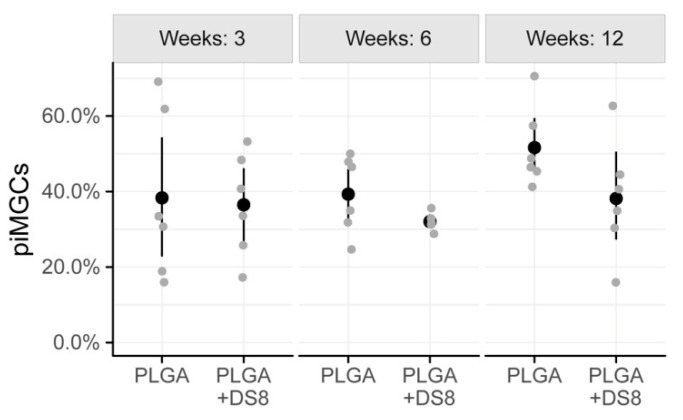
Scatter plot of peri-implant multi-nucleated giant cells (piMGCs), i.e., the percentage of the implant surface that is surrounded by MGCs. (Black dots refer to mean values of the respective groups.)

The percentage of the implant surface that was surrounded by MGCs showed high variation. After 3 weeks, 38.3% (SD 22.2) and 36.5% (SD 13.7) of the implant surface was covered by MGCs in the PLGA and PLGA+DS group, respectively. After 6 weeks, piMGCs was 39.3% (SD 10.3) in the PLGA group and 32.0% (SD 2.5) in the PLGA+DS8 group. After 12 weeks, piMGCS was 51.6% (SD 10.7) in PLGA and 38.2% (SD 15.6) in the PLGA+DS8 group (Figure 7).

In the PLGA groups, piMGCs tended to increase over time from 38.3% to 51.6%, whereas it remained rather constant around 38% in the PLGA+DS8 groups. However, this time-dependent change was not statistically significant (*p* = 0.212), probably due to the high variation and the low number of specimens. Likewise, the differences between the groups were not statistically significant at any time point (*p* = 0.097).

#### 3.3.4. Implant Diameter (I.DM)

**Figure 8:**
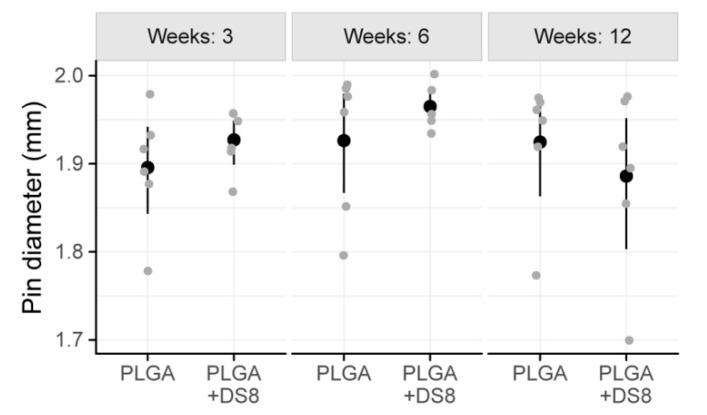
Scatter plot of the diameter of the implants (I.Dm). (Black dots refer to mean values of the respective groups.)

The mean diameter of the PLGA rods -being a measure for structural integrity or degradation- ranged from 1.89 mm to 1.97 mm. In detail, after 3 weeks I.DM was 1.90 mm (SD 0.07) in the PLGA group and 1.93 mm (SD 0.03) in the PLGA+DS8 group. After 6 weeks, I.DM was 1.93 mm (SD 0.08) and 1.96 mm (SD 0.02), and after 12 weeks, 1.92 mm (SD 0.08) and 1.89 mm (SD 0.10) in the PLGA and the PLGA+DS8 group, respectively (Figure 8).

There was no significant difference between the PLGA and the PLGA+DS8 groups at any time point (*p* = 0.657). No significant time-dependent change of I.DM was found either (*p* = 0.323). Accordingly, the degradation of the SR-PLGA implants was comparatively low in all groups independent of type and time of treatment.

## 4. Discussion

Inflammation is a naturally occurring response to injury [50,51,52]. The idea to modulate the immune reaction in the host bone in order to influence inflammation and bone regeneration is based on the close interplay of the immune system and bone metabolism. While the short initial acute inflammatory response is critical for bone healing and regeneration, a prolonged, unregulated chronic inflammation is detrimental and has destructive effects on bone tissue. Such chronic inflammation occurs as a reaction to implanted foreign materials [52]. The exogenous modulation of the natural immune response is intended to reduce the risk of chronic inflammation without interfering with the initial, critical acute inflammation phase of wound healing [52,53,54,55].

The aim of the present study was to evaluate the impact of DS containing poly(lactic-co-glycolide) implants on local tissue reactions in the femur of rats. The local application of the anti-inflammatory agent DS is an attempt to reduce excessive inflammation and thus, the risk of an FBR directly at the implantation site. Concurrently, the anti-inflammatory agent must not interfere with the critical initial acute inflammation phase ensuing (bone) injury in order to avoid negative effects on bone healing. Therefore, special emphasis was put on the impact of local DS releasing implants on bone regeneration.

In fact, we found MGCs characteristic for a foreign body reaction at the surface of the implanted PLGA rods of all groups. In rats treated with PLGA implants having no DS, FBR in terms of MGCs tended to increase over time, whereas in the groups treated with PLGA+DS, implants, piMGCs remained rather constant over the healing period of 12 weeks, which is indicative of an anti-inflammatory effect of DS on the tissues at the implant interface. However, the power of the statistical analysis is limited due to the small size of the experimental groups and failed to demonstrate statistical significance.

In fact, the role of MGCs present in the implantation bed of biomaterials is a controversially discussed issue in literature [56]. Although foreign body MGCs are traditionally associated with destructive inflammatory processes such as chronic inflammation, osteolysis, and disrupted bone healing, they do not necessarily have a negative effect on the healing process of the bone [57]. In contrast, these inflammatory cells appear to be critical for the degradation process of biomaterials via phagocytosis [26,58,59]. It is speculated that different subtypes of MGCs might drive the reaction to biomaterials into a rather short, inflammatory direction or in a more persistent way towards classical FBR and encapsulation [42]. There is increasing evidence that MGCs with an “M2-macrophage” phenotype are capable to promote angiogenesis and wound healing [60]. However, very fast degradation of some materials as well as the presence of a very high number of MGCs during the early phase of healing, are assumed to be detrimental and lead to fibrous encapsulation [61].

The present finding that MGCs are reduced by the presence of DS releasing PLGA rods in rat femora is in line with other studies showing suppression of inflammation through the application of DS loaded polymers. An in vitro study of Sidney et al. demonstrated that the local release of DS from PLGA scaffolds reduces cytokine-induced production of PGE2 in an osteoblast inflammation model [62]. Kim et al. investigated sutures incorporating PLGA nanoparticles, which were loaded with DS and decorated with polyethylene glycol and macrophage-targeting ligand mannose. It was shown that inflammation in macrophage culture and in an excisional wound healing model in the rat was significantly reduced compared to no-DS groups: In vitro, prostaglandin E2 synthesis was decreased when macrophages were treated with the sutures for 6 h. This effect was confirmed in vivo by showing less inflammation of the sutured tissues.

While the concept of loading polymers with DS is promising in reducing inflammation and thus, reducing the risk of foreign body reactions, the effect of DS on bone regeneration is disputed [63,64]. It is known that bone injury increases the local concentration of prostaglandins E and F [65]. DS being a non-selective COX inhibitor, however, inhibits the synthesis of prostaglandins, which may have negative affect osteoblasts and osteoclasts [66]. NSAIDs were demonstrated to reduce the proliferation, differentiation, adhesion and migration of osteoblasts and osteoclasts, matrix maturation and bone mineralization [67,68,69,70]. Particularly in the very early stage of bone healing, diclofenac decreases the number of osteoblasts at the bone defect site and bone mineral density [68].

Yet, there is evidence that the negative effect on bone regeneration might be dose-dependent and even reversible [71,72]. When NSAIDs are applied systemically, the excessive suppression of inflammation via DS is associated with delayed fracture healing and an increased risk of non-union after long-bone fractures [73,74,75]. The local administration of anti-inflammatory agents might circumvent side-effects that occur due to a systemically administered higher dosage since a lower dose of the drug is needed to achieve the desired effect at the target tissue, i.e., the site of injury [76].

Accordingly, the second focus of this study was laid on the regenerative capacity of bone when DS is released locally. In the medullary cavity of all rats’ femora, the implantation of PLGA and PLGA+DS8 rods induced the formation of a layer of newly formed bone islands parallel to the contour of the implants. This peri-implant bone capsule surrounded the implants and increased over the treatment period of 12 weeks. DS released by the PLGA rods seemed not to have a negative impact on the bone regeneration. These findings suggest that the local release of DS by biodegradable PLGA implants might be capable to reduce inflammatory reactions associated with the increased presence of MGCs without affecting bone regeneration in the femora of rats.

A critical aspect in this context is the temporal pattern of drug release which is related to the rate of hydrolytic/enzymatic degradation of the polymer. Simon and O’Connor reported time-dependent effects of NSAID in a fracture model in the rat [77]. While the administration prior to fracture or two weeks later, did not affect fracture healing negatively, NSAID administration in the initial inflammatory phase was associated with reduced callus strength and increased incidence of non-union [77]. This effect, however, seems to be reversible as demonstrated by a study in rats given a COX-2 selective inhibitor for 21 days after femoral fracture. Two weeks after drug administration was stopped the rate of non-unions dropped to a level similar to that seen in the control group [78].

The 80/20 PLGA rods used in this study showed only very slight evidence of degradation: Twelve weeks after implantation, the structural integrity of the implanted PLGA rods was maintained. The diameter of the rods did not decrease significantly within the treatment period. Microscopic pits and fissures as well as a ridged, slightly fringed surface of rods were observed in most specimens. The low degradation was expected since a ratio between PLA and PGA was 80:20. The degradation rate of PLGA implants can be controlled by varying the ratio between the glycolic and lactic acid components [79]. Glycolic acid is more hydrophilic than lactic acid which leads to more rapid water uptake and thus to faster hydrolytic degradation compared to the more hydrophobic lactic acid. Accordingly, PLGA devices rich in glycolide degrade faster than lactide rich PLGAs [11,12,79].

PLGA 80:20 miniscrews, for example, are reported to take over 18 months to degrade in the cranial bone of rabbits [80]. In the patient, 85:15 PLGA osteosyntheses implants were described to degrade within 12 months [28,81].

From a clinical point of view, polymeric implants are supposed to provide stability at the beginning and to allow gradual load transmission to healing bone while they degrade [10]. Therefore, slow degradation of polymeric implants is highly beneficial in many orthopaedic settings. The addition of DS to the PLGA production had no influence on the degradation properties of the implants.

The release of drugs from polymers is a very complex and dynamic process. Besides hydrolysis and degradation of polymers, the anatomical implant site and its microenvironment (including local pH, diffusion, osmotic pumping) are also decisive [82,83]. Unfortunately, the experimental setting of this study does not allow drawing final conclusions about the influence of polymer degradation on drug release nor identifying other factors that may affect drug release.

A limitation of this study is the small group size which results in a relatively low statistical power. Our data suggest that DS containing PLGA implants tend to reduce inflammation in terms of MGCs in their immediate proximity without impairing new bone formation. However, these results have to be taken with caution and need to be validated in a larger sample. Another drawback of this study is that the histological thin-ground technique applied here does not allow immunohistochemical characterization of cells. The histological technique used in this study is a well-established method to produce un-decalcified thin ground sections [40]. One apparent advantage over paraffin histology is that the tissue samples are not demineralized before embedding. The samples are embedded into a light-curing resin which hardens and allows being cut and ground without altering the appearance of tissues: There is no shrinkage, distortion or deformation during the production of the histological thin ground sections as it is known from paraffin sections where the implant is also often ripped out by the microtome knife. This is particularly important when quantifying tissues with histomorphometric means. We chose the non-decalcifying technique because we wanted to identify and accurately measure the effect on bone healing in the first place. Further research is needed to characterize the cells at the implant-tissue interface by means of immunohistochemistry.

In future studies, a prolonged observation period and implants with different DS release patterns would increase our understanding of the therapeutic effect of DS containing SR-PLGA implants. Many studies analyzing the degradation of polymeric implants and associated tissue regeneration focus on the histomorphometric quantification of tissues involved in the healing process. The present study complements the information gained by using histomorphometry, by having a detailed histological description of the host tissues: In the long term, FBR is commonly described to result in the infiltration of fibroblasts and the formation of granulation tissue that results in the formation of a fibrous capsule around the implant [21,22]. In this way, the implanted biomaterials become separated from the surrounding tissues [21,22]. Carnicer-Lombarte et al. (2019) reported that a mismatch between the mechanical properties of an implant and of the host tissue is a driving force for FBR and fibrotic capsulation of biomaterials. When stiff implants are coated with a thin layer of hydrogel or silicone the elastic modulus of the implant surface can be reduced to a tissue-like level. This, in turn results in decreased fibrosis and inflammation in the host tissue [84].

In the current study, we did not find a fibrous tissue encapsulation, but a layer of newly formed “bone islands” that lined the surface of the implant like a fenestrated osseous capsule. Since the bone islands were only very sporadically in direct contact with the biomaterial, new bone formation might not be interpreted as a process of “osseointegration” comparable to the osseointegration of endosseous implants. Bosshardt et al. (2011) reported that the bone debris originating from the preparation of an implant site through drilling contributes to the induction of bone formation at the surface and in the vicinity of implants [85]. It appears that the local bone trauma produced by the surgery might have triggered wound healing and bone regeneration not only in the injured intercondylar region where the drill hole was produced but also in the proximal direction, in the marrow cavity. Along with the individual immune situation and response, biomechanical factors might also determine whether a fibrous tissue encapsulation or an osseous covering will form [86]. It remains to be elucidated if and to what extent the viscoelastic properties of the implants may lead the way to either fibrous or osseous encapsulation.

As a part of FBR, numerous MGC of various sizes and shapes were identified on the surface of the implant. In contrast to newly formed bone islands, MGCs were in direct contact with the PLGA surface. Frequently, they were found in pits of the implant surface, resembling resorption lacunae of the bone. The characterization of MGCs in the rat bone, including their distribution, frequency and size provides interesting insight into tissue reactions to PLGA implantation in the rat. As demonstrated by other researchers, FBR to biodegradable biomaterials may differ significantly between different species and even between different strains of the same species [87,88,89]. Therefore, detailed characterization of FBR provides the basis for the choice of animal model and the interpretation of tissue reactions which all together contribute to a better understanding of inflammatory processes at the tissue level.

## 5. Conclusions

This study analysed the impact of local DS releasing PLGA rods on bone regeneration and inflammation in the direct vicinity of implanted rods. The implantation of PLGA and PLGA+DS8 rods induced the formation of a layer of newly formed bone islands parallel to the contour of the implants. PLGA+DS8 rods tended to reduce the presence of multi-nucleated giant cells (MGCs) associated with FBR at the implant surface. Although the systemic administration of DS is known to be associated with compromised bone healing, the local release of DS via PLGA rods did not have negative effects on bone regeneration in the femora of rats throughout the study that extended for 12 weeks. Future studies with a longer follow-up period are; however, needed to draw conclusions about the long-term impact on inflammation and bone healing when the degradation of the implants proceeds.

## Figures and Tables

**Table 1:** piBC—test for time differences within the poly(lactide-co-glycolide) (PLGA) and the PLGA+DS8 groups. (* indicates statistical significance at *p* < 0.05.).

Treatment Group	Follow-up Period	Difference	Std. Error	*p*-Value	Statistical Significance
PLGA	3 weeks vs. 6 weeks	−0.19	0.06	0.0110	*
PLGA	3 weeks vs. 12 weeks	−0.20	0.06	0.0068	*
PLGA	6 weeks vs. 12 weeks	−0.01	0.06	0.9999	
PLGA+DS	3 weeks vs. 6 weeks	−0.11	0.07	0.3902	
PLGA+DS	3 weeks vs. 12 weeks	−0.24	0.06	<0.001	*
PLGA+DS	6 weeks vs. 12 weeks	−0.13	0.06	0.1502	

## Supplementary Materials

Available online at https://www.mdpi.com/2072-666X/11/12/1098/s1.
