# Supplementary material for: The Effect of Diclofenac Sodium-Loaded Poly(Lactide-co-Glycolide) Rods on Bone Formation and Inflammation: A Histological and Histomorphometric Study in the Femora of Rats
Source: Micromachines (Basel). 2020 Dec 12;11(12):1098. doi: 10.3390/mi11121098 (PMC7764049; doi:10.3390/mi11121098)
Supplement: Supplementary file 1 [file micromachines-11-01098-s001.pdf]

The Effect of Diclofenac Sodium-Loaded PLGA Rods on Bone Healing and Inflammation: A Histological and Histomorphometric Study in the Femur of Rats

Karoline M. Reich, Petrus Viitanen, Ehsanul Hoque Apu, Stefan Tangl
Nureddin Ashammakhi

ad **2.3.2 Histomorphometry**

SEGMENTATION ALGORITHM:

The morphometric software Definiens Developer 6.0.3®/Definiens Developer XD 2.7® (Definiens AG, Munich, Germany) was used to automatically segment and classify the different tissue types within the ROI. Therefore, an algorithm was developed considering the shape and color of all segments as well as the relationship to other neighboring segments. In this way, old and newly formed bone, cartilage, soft/fibrous tissue, PLGA rod and background could be identified.

- Software
  - Definiens Developer XD 2.7
- Input
  - Histological image
  - Manually drawn mask for the different regions

1. Region mask is thresholded and applied to histology
2. Masked histology image is downsampled to 50%
3. Classify as Bone areas where R-G ≥ 20 and rest as Void
4. Create a 5-pixel wide border between Void and Bone, classify as Void where R-G ≤ 10
5. Loop while something changes:
   a. Grow Void by 1 pixel into remaining border region all are true:
      i. R-G ≤ 0
      ii. More than 40 % Void in a 5x5 region centered on candidate pixel
   b. Grow Bone by 1 pixel into remaining border region all are true:
      i. R-G ≥ 50
      ii. More than 40 % Bone in a 5x5 region centered on candidate pixel
6. Repeat above loop with thresholds R-G ≤ 10 for Void and R-G ≥ 50 for Bone
7. Repeat above loop with thresholds and density criteria R-G ≤ 20, 45% for Void and R-G ≥ 20, 45% for Bone
8. Repeat above loop with thresholds and density criteria R-G ≤ 20, 35% for Void and R-G ≥ 20, 35% for Bone
9. Repeat above loop without thresholds and density criteria of 60% reducing the criteria by 10% each iteration until the border region is completely filled
10. Copy segmentation to full resolution
11. Loop 3 times:
    a. Grow Void by 1 pixel into remaining border region if more than 50 % Void in a 5×5 region centered on candidate pixel

b. Grow Bone by 1 pixel into remaining border region if more than 50 % Bone in a 5×5 region centered on candidate pixel

Explanatory comments:

[3]*R-G (Difference between red and green layers of the RGB image)*

[4]*The border region is created because in this region misclassifications are the most laborious to correct. By iteratively growing Void and Bone with decreasingly restrictive thresholds and density criteria, a more accurate and smooth border between both classes is found.*

[9]*This final loop guarantees that the entire border region is filled with either Bone or Void classes.*

[11]*The class borders are not smooth due to the upscale. In this loop, the border is smoothed.*

Based on this segmentation procedure, the remaining falsely classified areas were manually corrected under microscopic control using Adobe Photoshop software (Adobe, San Jose, CA, USA).
